# Generalist in allogeneic hematopoietic stem cell transplantation for MDS or AML: Epigenetic therapy

**DOI:** 10.3389/fimmu.2022.1034438

**Published:** 2022-10-04

**Authors:** Guancui Yang, Xiang Wang, Shiqin Huang, Ruihao Huang, Jin Wei, Xiaoqi Wang, Xi Zhang

**Affiliations:** ^1^ Medical Center of Hematology, Xinqiao Hospital, Third Military Medical University (Army Medical University), Chongqing, China; ^2^ State Key Laboratory of Trauma, Burns and Combined Injury, Third Military Medical University (Army Medical University), Chongqing, China; ^3^ Department of Hematology, Affiliated Hospital of North Sichuan Medical College, Nanchong, China

**Keywords:** hematopoietic stem cell transplantation, epigenetic therapy, graft-versus-host disease, myelodysplastic syndrome, acute myeloid leukemia

## Abstract

Allogeneic hematopoietic stem cell transplantation (allo-HSCT) remains the only curative treatment for patients with myeloid malignancies such as myelodysplastic syndrome (MDS) and acute myeloid leukemia (AML). However, relapse and graft-versus-host disease (GvHD) still affect the survival of patients who receive allo-HSCT, and more appropriate therapeutic strategies should be applied at all stages of transplantation to prevent these adverse events. The use of epigenetics agents, such as hypomethylating agents (HMAs), has been explored to decrease the risk of relapse by epigenetic modulation, which is especially effective among AML patients with poor mutations in epigenetic regulators. Furthermore, epigenetic agents have also been regarded as prophylactic methods for GvHD management without abrogating graft versus leukemia (GvL) effects. Therefore, the combination of epigenetic therapy and HSCT may optimize the transplantation process and prevent treatment failure. Existing studies have investigated the feasibility and effectiveness of using HMAs in the pretransplant, transplant and posttransplant stages among MDS and AML patients. This review examines the application of HMAs as a bridge treatment to reduce the tumor burden and the determine appropriate dose during allo-HSCT. Within this review, we also examine the efficacy and safety of HMAs alone or HMA-based strategies in posttransplant settings for MDS and AML. Finally, we provide an overview of other epigenetic candidates, which have been discussed in the nontransplant setting.

## Introduction

Allogeneic hematopoietic stem cell transplantation (allo-HSCT) is the only curative treatment for most patients with MDS or AML (1, 2). However, relapse remains the primary cause of mortality in patients receiving allo-HSCT for MDS or AML (3, 4). Epigenetic agents, mainly including hypomethylating agents (HMAs) and histone deacetylase inhibitors (HDACi), have become widely used in the pretransplant, transplant, and posttransplant stages for MDS or AML (5–9) and are regarded as candidates to reduce the tumor burden, as they have tolerable toxicity and mitigate graft-versus-host disease (GvHD) without influencing graft versus leukemia (GvL) (9, 10). Furthermore, HMAs have been proven effective in exerting an antitumor effect by promoting cell differentiation and apoptosis in leukemia cells (11, 12) and by reactivating silent tumor suppressor antigens like MAGE-1 and WT-1 to improve the response to other therapeutic hematologic malignancies (10, 13, 14). In this review, we will summarize the application of epigenetic agents in the pretransplant, transplant and posttransplant stages, including bridging treatment, preconditioning regimens, maintenance, preemptive therapy, and salvage therapy.

## Pre-HSCT

Minimal residual disease (MRD) before transplantation significantly increases the probability of relapse after transplantation, which affects patient outcomes (15). Thus, some patients need bridging treatment to control the disease status. HMAs, including azacytidine (AZA) and decitabine (DAC), are widely used in bridge therapy before transplantation.

The safety and efficacy of HMAs has been confirmed by several studies and included in the guideline for HSCT-based treatment of AML (16). One retrospective study reported that compared with a non-DAC-based regimen, patients bridging with DAC showed a higher pretransplant bone marrow complete response (CR) and longer 2-year overall survival (OS) (17). A phase II multicenter study by VOSO et al. enrolled 97 patients with myeloid neoplasia for whom bridging therapy with AZA was administered before transplantation (18). The results showed that the median survival time was 15.2 months, and the overall response rate (ORR) reached 38% (**Table 1**). In another multivariate analysis, response to AZA was the only independent prognostic factor for survival. Xu et al. retrospectively analyzed the role of HMAs in bridging treatment of intermediate-risk AML. The treatment regimen was DCAG (DAC, cytarabine, aclarubicin hydrochloride and granulocyte colony-stimulating factor) or standard “3+7” regimen with or without HSCT. The results showed that DCAG-bridged HSCT had the best prognosis (5-year OS: 80%, EFS: 85.7%) (23). Therefore, bridging allo-HSCT with HMAs before transplantation can benefit patients. Additionally, the application of HMAs could also increase the safety of transplantation. A retrospective study by Kim et al. demonstrated benefits in NRM after bridging therapy in patients with high bone marrow counts (>5%) (19). However, retrospective trials reported that HMAs could not improve OS/PFS or decrease the relapse rate (21, 22). For elderly individuals, HMAs have been the standard therapy because of their good tolerance (16).

**Table 1:** The use of hypomethylating agents prior to hematopoietic stem cell transplantation in MDS or AML.

Intervention strategy	Agent	Patient	Efficacy		Safety		Ref
			OS/Median OS	ORR (CR, PR)	aGvHD	cGvHD
**Bridge**	HMA	109 (81 vs 28)	17-m vs 15-m	19.4% (13.4%, 6%)	17.3% vs 17.9% (II-IV)	21.0% vs 32.1% (extended)	(19)
	HMA	98	4-y: 53.8%	CR: 12.2%	29.9% (II-IV)	48.1%	(20)
	AZA	97	15.2-m	38% (24%, 14%)	6% (III-IV)	29%	(18)
	HMA	209 (77 vs 132)	3-y: 42% vs 41%	CR (32.5% vs 68.2)	N/A	N/A	(21)
	AZA	163 (98 vs 48 vs 17)	3-y: 55% vs 48% vs 32%	CR (80.6% vs 66.7% vs 52.4%)	N/A	N/A	(22)

HMA, hypomethylating agents; AZA, Azacytidine; OS, overall survival; ORR, overall response rate; CR, complete remission; PR, partial remission; aGvHD, acute graft-versus-host disease; cGvHD, chronic graft-versus-host disease; N/A, not available.

For patients with different disease states, the bridging scheme and the timing of transplantation should be different. For minimal residual disease (MRD) negative patients, HSCT should be conducted as soon as possible. It is currently believed that a shorter disease duration pre-HSCT is associated with improved OS and disease-free survival (DFS) and decreased treatment-related mortality (TRM) (24, 25). Patients with bone marrow counts greater than 5% and those with CR but MRD^+^ may benefit from bridging treatment (26–28). For this kind of patient, HMAs can reduce the tumor burden, achieving the purpose of “debulking” and improving the prognosis of patients. Yahng et al. conducted a retrospective study of patients with a blast count > 5% who underwent HMA bridging treatment and showed that the prognosis of the bone marrow response group at the time of transplantation was better than that of the no bone marrow response group (20). Sungwoo park’s study showed that some patients with SD also benefited from the use of HMAs (29). Furthermore, numerous studies have shown that even if patients achieve CR, MRD at the time of transplantation is an independent predictor of subsequent relapse (30–33). It has been reported that oral 5-AZA, compared with placebo, can help AML patients to prolong the time of MRD^-^ (11 months vs. 5 months), and the rate of MRD^+^ to MRD^-^ conversion was higher with oral azacitidine (15). With the continuous development of detection technology for MRD, this indicator is being evaluated before HSCT. However, it remains unclear whether patients who achieved CR but did not achieve MRD^-^ can benefit from bridging treatment before transplantation.

## During HSCT

As an epigenetic agent, DAC has been widely used in a variety of condition regimens due to its low toxicity and antitumor effect. For the application of DAC in condition regimens, the focus of general attention is the dose. This has gone through a process of gradual optimization from high to low doses. As early as 2003, the de Lima team used high-dose DAC (100 mg/m^2^ every 12 hours×4, 150 mg/m^2^ every 12 hours×4, 200 mg/m^2^ every 12 hours×4) combined with a myeloablative condition regimen (34). Twenty-three patients with myeloid tumors were included in this retrospective study, and the median survival reached 17.5 months, but the higher treatment-related mortality rate deserves attention (3 years: 35%). Therefore, a series of clinical trials of low-dose DAC were carried out, including a 10-day regimen and a 5-day regimen. A phase II randomized clinical trial showed that both the 5-day regimen and the 10-day regimen were effective in elderly patients with AML, and there was no significant difference (35) (**Table 2**). We can observe that the 10-day regimen increases the risk of infection while prolonging survival and reducing relapse, which may be related to the toxicity of DAC (39, 42). The use of DAC doses varies from country to country. For example, in China, considering the patient’s physique, more 5-day DAC schemes are used. Overall, we found that low-dose DAC combined with pretreatment can reduce the proportion of TRM after transplantation, prolong survival and reduce GvHD. A retrospective study from China analyzed 236 AML patients, of whom 59 received DAC combined with the Bucy condition regimen and 177 received the Bucy condition regimen without DAC (37). The results showed that the 2-year OS rate of patients in the DAC group was significantly improved, and the TRM rate within 100 days was 0%. A retrospective study reported by Wang et al. included 76 patients with AML or MDS (38). Forty patients received DAC combined with the Bucy/BuFlu condition regimen, and 36 patients only used the Bucy/BuFlu condition regimen. The results showed that the incidence of grade III-IV acute GvHD decreased significantly. A retrospective study by Li et al. compared the effects of short-term and high-dose DAC (75 mg/m^2^ on day -9 and 50 mg/m^2^ on day -8) with short-term and low-dose DAC (15 mg/m^2^/day on days -10 to -8) on the prognosis of patients (36). The results showed that the three-year overall survival rate of the high-dose group was better than that of the low-dose group, while the incidence rates of relapse, acute GvHD and chronic GvHD in the higher-dose group were lower than those in the low-dose group. Although patients generally benefit from the addition of HMAs to conditioning regimens, the optimal dose is still unclear and needs to be further explored.

**Table 2:** The use of hypomethylating agents during hematopoietic stem cell transplantation in MDS or AML.

Intervention strategy	Agent	Dose	Patient	Efficacy		Safety		Ref
				OS/Median OS	Relapse/ORR	aGvHD	cGvHD
**Condition**	DAC+MAC	1) 75 mg/m^2^×1d; 50 mg/m^2^×1d 2) 25 mg/m^2^/day×3d 3) none	65 (20 vs 18 vs 27)	3-y: 50% vs 22.2% vs 18.5%	3-y: 41.1% vs 74.6% vs 88.1%	0% vs 11.1% vs 22.2% (III-IV)	5% vs 44% vs 37%	(36)
	DAC+BuCy	20 mg/m^2^/d×5d	236 (59 vs 177)	2-y: 80.7% vs 43.5%	8-m vs 5-m	10.2% vs 12.4% (III-IV)	39% vs 32.2%	(37)
	DAC+BuCy/BuFlu	15 mg/m^2^×5d	76 (40 vs 36)	no difference	7-m vs 3-m	12.4% vs 41.5% (II-IV)	17.5% vs 25%	(38)
	DAC+MAC	20 mg/m^2^/d×10d	20	100d: 65%	N/A	27.80% (III-IV)	1-y: 40%	(39)
	DAC+MST	20 mg/m^2^/d×5d	22 (11 vs 11)	24-m vs 14.3-m	81.8% vs 54.5%	N/A	N/A	(40)
	DAC+MST	25 mg/m^2^/d×4d	21 vs 22	2-y: 84.7% vs 34.1%	81% vs 50%	N/A	N/A	(41)

DAC, decitabine; MAC, myeloablative conditioning; MST, micro-transplantation; OS, overall survival; ORR, overall response rate; aGvHD, acute graft-versus-host disease; cGvHD, chronic graft-versus-host disease; N/A, not available.

Huisheng Ai et al. combined DAC with micro transplantation to treat MDS/tAML patients. The overall response rate was 65.1%, and the overall response rate in the MDS group was significantly higher than that in the tAML group (41). The report by Li et al. also showed that DAC combined with MST could increase the overall response rate (40). This is a new scheme of DAC combined with micro-transplantation (MST), which can effectively prolong the survival of patients with high-risk MDS and TAML, especially for MDS patients, and may become an effective bridging method for allogeneic transplantation. Overall, the combination of HMAs with MST results in an increased overall response rate in patients, and the benefit is better in patients with MDS than in those with AML.

## Post-HSCT

### Maintenance therapy

Recently, a phase 3, open label, randomized study (43) compared AZA maintenance vs. observation after transplant in high-risk MDS and AML patients. The observations were noteworthy in that both PFS and OS showed no significant difference between the two arms, which differed from the previous encouraging results (44–46). A probable cause for the outcome was that the patient population included in this study was heterogeneous (the majority of enrolled patients had high-risk AML) and that the natural history of AML and MDS was different, with the former having a higher incidence of an adverse prognosis (47–49). Multivariate analyses also suggested that AZA could provide more clinical benefit in patients with MDS than in those with AML. In addition, grade 3 or higher drug-related AEs were observed in the majority of patients in the study, including bone marrow suppression, liver toxicity and infection. Therefore, the toxicity of AZA should be considered. Compared with AZA, DAC might be safer as a posttransplant maintenance treatment for AML or MDS because of its very low nonhematologic toxicity (50). In a phase I/II study (51), the MTD was not reached, but relatively lower doses (10 mg/m^2^×5 days) seemed to be a better tolerated dose for DAC maintenance in the post-HSCT setting. CC-486, the oral formulation of AZA, is more likely to be associated with better OS and lower GvHD risk (**Table 3**) (52), which might be related to the means of administration. Oral AZA could lead to extended dosing to prolong AZA activity and better compliance (71, 72). Currently, several studies have suggested that remission duration is one of the strongest predictors of posttransplant survival (73, 74); therefore, a long CC-486 remission duration could reduce the risk of relapse posttransplant. Thus, the use of oral AZA instead of injectable AZA could be explored in future studies in AML and MDS posttransplantation. Overall, MDS/AML patients receiving HMA maintenance after allo-HSCT had better outcomes as well as a reduced incidence of chronic GvHD, consistent with an updated systematic review and meta-analysis (75). Panobinostat (PNB, a histone deacetylase inhibitor), due to its immunomodulatory activity (76) that may enhance leukemia-specific cytotoxicity and mitigate GvHD, was also applied in a transplant setting. The safety and efficacy of PNB was explored in a phase I/II clinical trial (53) in patients with high-risk MDS/AML, and it was well tolerated, with a relatively low relapse rate and incidence of GvHD. Another HDACi, vorinostat, was also effective in GVHD prevention by reducing proinflammatory cytokines and increasing regulatory T-cell number and function after allo-HSCT in MDS and AML patients (9).

**Table 3:** The use of hypomethylating agents after hematopoietic stem cell transplantation in MDS or AML.

Intervention strategy		Agent	Patient	Efficacy		Safety		Ref
				OS/Median OS	RFS/ORR (CR, PR)	aGvHD (grade 3-4)	cGvHD (moderate or severe)
**Maintenance**	Mono	AZA	87 vs 93	2.52-y vs 3.56-y^	2.07-y vs 1.28-y^	4.3% vs 2.1%^	25.8% vs 30.8%^	(43)
		DAC	22	2-y: 56%	2-y: 48%	9.1%	54.5%	(51)
		CC-486	30	1-y: 81%	1-y: 72%	3%	10%	(52)
		PNB	42	2-y: 81%	2-y: 75%	7.1%	29%	(53)
	Combination	AZA+DLI	30 vs 58	2-y: 65.5% vs 63.3%^	2-y: 65.5% vs 51.5%^	16.70%	13.30%	(54)
		DAC+rhG-CSF	100 vs 102	2-y: 85.8% vs 69.7%	2-y: 81.9% vs 60.7%	N/A	23.0% vs 21.7%^	(55)
		DAC+VEN	20	2-y: 85.2%	2-y: 84.7%	55%*	20%*	(48)
		AZA+APR-246	43	1-y: 79%	1-y: 60%	18%*	12%	(56)
		DAC+PNB	110	2-y: 50%	2-y: 49%	5%	22%	(57)
**Pre-emptive**	Mono	AZA	20	1-y: 50%	80% (50%, 30%)	N/A	N/A	(58)
		AZA	53	1-y: 75%	1-y: 46%	N/A	N/A	(59)
**Salvage**	Mono	AZA	39	2-y: 25%	30.7% (7.7%, 23.0%)	10.3%	N/A	(60)
	Combination	AZA+DLI	30	117 days	30% (23%, 7%)	10%	17%*	(61)
		AZA+DLI	49	6-m	22.4% (20.4%, 2.0%)	5.1%*	12.5%*	(62)
		AZA+DLI	154	2-y: 29%	33% (27%, 6%)	23%	27%	(63)
		DAC+DLI	36	2-y: 11%	25% (17%, 8%)	19%*	5%*	(64)
		DAC+DLI	26	4.7-m	19.2% (15.4%, 3.8%)	3.8%	0	(65)
		AZA+LEN	29	27-m vs 10-m (responded vs nonresponders)	47% (40%, 7%)	7%	N/A	(66)
		AZA+GO	50	11-m in CR group	CR: 24%	N/A	N/A	(67)
		AZA+NIVO	70	6.3-m	33% (22%, 11%)	N/A	N/A	(68)
		HMA+VEN	32	3.7-m	47% (36.7%, 10%)	10%*	3.3%*	(69)
		HMA+VEN	44	8.1-m vs 2.8-m (CR/CRi vs non CR/CRi)	38.6% (34.1%, 4.5%)	N/A	N/A	(70)

^, not significant difference; *, all grade aGvHD or cGvHD; AZA, azacytidine; DAC, decitabine; PNB, panobinostat; DLI, donor lymphocyte infusion; rhG-CSF, recombinant human granulocyte colony-stimulating factor; VEN, venetoclax; HMA, hypomethylating agents; LEN, lenalidomide; GO, Gemtuzumab Ozogamicin; NIVO, nivolumab; OS, overall survival; RFS, relapse-free survival; ORR, overall response rate; CR, complete remission; PR, partial remission; aGvHD, acute graft-versus-host disease; cGvHD, chronic graft-versus-host disease; N/A, not available.

For combined therapies, a phase II randomized controlled trial (RCT) from China evaluated the efficacy of low-dose DAC (5 mg/m^2^×5 days) maintenance combined with recombinant human granulocyte colony-stimulating factor (rhG-CSF) after allo-HSCT in AML or MDS patients (55). The results showed that this treatment reduced the relapse rate due to the GvL effect, which was generated by the increased numbers of NK and CD8^+^ T cells, and controlled the progression of GvHD due to the increased numbers of Treg cells. Another single-arm study by Burak Kalin et al. (57) showed that PNB after allo-HSCT alone or in combination with low-dose DAC (10 mg/m^2^×3 days) was feasible in AML patients, especially in low-risk AML patients. Low-dose DAC (15 mg/m^2^×3 days) plus venetoclax (BCL-2i) has also shown promising efficacy in maintenance therapy after HSCT (48). Together with an acceptable toxicity profile, these data suggest that low-dose DAC in combination with traditional therapies may exert additive or synergistic effects in AML or MDS maintenance therapy posttransplantation. Furthermore, HMAs could also be combined with targeted drugs as maintenance therapy posttransplant. A phase II trial presented at the 2021 annual meeting of the American Society of Hematology (ASH) suggested that a novel targeted agent, eprenetapopt (APR-246, a small molecule p53 stabilizer), in combination with AZA as maintenance therapy for TP53-mutated AML or MDS after allo-HSCT was relatively favorable and well tolerated (56). APR-246/AZA was proven to reactivate the p53 pathway and induce apoptosis in tumor cells (77); thus, TP53-mutated MDS/AML may be better targeted by this combination to conventional treatments. Moreover, AZA in conjunction with gemtuzumab ozogamicin (GO), which is associated with a long survival (>700 days), could be safely administered as maintenance therapy in MDS and AML patients after HSCT (78).

### Salvage therapy and preemptive therapy

The prognosis of AML and MDS patients who relapse after allo-HSCT is usually poor, with a 3-year OS of 19% (79). Thus, relapse is a major cause of treatment failure in patients receiving allografts (80). Only 20% of patients respond to traditional salvage chemotherapy or DLI (81, 82), and even after receiving a second transplant, the 5-year OS for these patients is lower than 30% (83). HMAs have also emerged in recent years as salvage treatment modalities in patients with MDS or AML. The outcome of HMA monotherapy was dismal in relapsed MDS or AML patients after allo-HSCT, with a 2-y OS of 25% (60), and combination therapies are often needed to effectively treat these patients.

Combined HMAs/DLI can not only reduce the incidence of GvHD, the major complication of DLI in the treatment of relapsed AML after allo-HSCT (approximately 40% of patients develop GvHD after DLI) (82), but also enhance the immunomodulatory role, such as increasing the response of CD8+ T cells (45, 84). A phase II study (61) of 30 patients demonstrated the feasibility and clinical efficacy of AZA in combination with DLI as salvage therapy for relapse after allo-HSCT. However, another recent prospective study of 49 patients showed that the administration of AZA and DLI had no significant impact on either response or survival (62). We evaluated the regimens of two different studies, and the latter administered DLI one day after AZA. Due to the cytotoxicity of AZA (85), the administration of DLI a relatively short period after AZA might exert a toxic effect on lymphocytes and decrease the GvL effect. Thus, it might be inappropriate to administer DLI immediately after AZA. Regarding DAC-DLI, a retrospective study (64) examined 36 AML and MDS patients who were treated with DAC and DLI for relapse after HSCT. More than half of the patients received other salvage therapies prior to DAC. As a result, 6 (17%) patients achieved CR, including one-third of patients with previous AZA-DLI failure due to AZA intolerability. Therefore, DAC-DLI could be an alternative to AZA-DLI or even a second choice after AZA-DLI failure. Another retrospective study (86) also agreed with the conclusion that DAC could result in durable survival in patients with more aggressive hematologic relapse when combined with DLI in AML or MDS after HSCT. Taken together, additional multicenter or prospective research with a larger population should be conducted in the future to compare AZA and DAC as salvage therapy in combination with DLI for relapse after HSCT, and multivariate analyses should be conducted to identify patients who may benefit most from the combination of DLI. Another combination agent was lenalidomide (LEN), which is known to cause myelosuppression and might eliminate the abnormal MDS/AML clone (87). Additionally, Len often causes acute GvHD after allo-SCT (88, 89), and thus, combined AZA/Len might theoretically improve clinical activity in relapsed AML or MDS without increasing the risk of severe GvHD. In the VIOLA trial, Charles Craddock et al. (66) found that AZA/Len therapy was well tolerated, and no severe GvHD was observed. Interestingly, the authors also found that this combination did not change the T-cell phenotype, which was previously reported to be associated with relapsed AML (90, 91). As a result, AZA in combination with Len could become a preemptive strategy in the post-allo-HSCT setting since it may not affect the results of the MRD assay. First, when combined with DLI/LEN, it has been shown to mitigate GvHD without sacrificing GvL. Second, HMAs have also been applied posttransplantation with some targeted drugs, such as Ven, GO, ICI (immune checkpoint inhibitor) and APR-246. AZA and Ven displayed combinatorial antitumor activity *in vivo* through transcriptional induction of the proapoptotic BH3-only protein NOXA (92). GO/HMA was associated with enhanced GO and anti-CD33-mediated inhibition of leukemia cell growth (93). HMAs increase the expression of PD-1 and PD-L1, especially in AML. APR-246/HMAs was demonstrated to reactivate the p53 pathway (77).

Venetoclax (VEN), a selective inhibitor of the antiapoptotic protein Bcl-2, has been demonstrated to have the potential to sensitize AML cells to HMAs (94). Recent studies have shown encouraging treatment effects in relapsed/refractory (R/R) AML and elderly patients with combined HMAs/VEN (95, 96), while the clinical value for HMAs/Ven in the transplantation setting has been explored preliminarily, and a phase 3, randomized clinical study of HMAs/VEN in AML posttransplantation (NCT04161885) is now ongoing. In a recent multicenter retrospective study from China (70), TP53 mutation (HR = 17.339, p =.033) and relapse within 1 year posttransplantation (HR = 6.261, p =.026) were identified as independent risk factors when HMAs/VEN was used in relapsed AML or MDS patients. The results from Esther Schuler et al. (69) suggested that HMAs/VEN combinations for molecular relapse or as first salvage therapy conferred better benefit.

There is now substantial evidence showing that patients who are molecular relapsed/hematological relapse with lower blast count or a longer interval from HSCT to relapse might be associated with better survival when HMAs-based therapies are used in relapsed AML or MDS (61–63, 69, 70, 97). Thus, for molecular relapsed (bone blast<5%) MDS or AML according to MRD determination, intervention strategies should be recommended and implemented as early as possible. Monotherapy with AZA has been suggested to delay relapse with a 1-y PFS of approximately 50% (59) as a preemptive treatment in higher-risk MDS or AML patients with MRD positivity after HSCT.

## Discussion

It has been shown that disease stage at the time of transplantation is one of the most important factors that influences outcome after allogeneic HSCT (32, 98), and a blast count greater than 5% is associated with relapse-related poor prognosis. Furthermore, it can be a timely process to find a human leukocyte antigen-matched donor after diagnosis. Disease progression and complications may be an obstacle to HSCT without bridging treatment. Therefore, it is feasible and essential to conduct bridge therapy prior to transplantation to reduce the tumor burden, but the trial results were not always good and remain to be further explored (99). In addition, the use of AZA can influence the mutation spectrum and evolution of new subclones after transplantation (100).

As a condition regimen, there are relatively few studies on the application of HMAs during transplantation. Patients may benefit more from conditioning regimens using HMAs, especially at a lower dose. Two regimens (5 days and 10 days) of low-dose commonly used therapies show fair efficacy. Compared with the 10-day regimen, a low incidence of infection was reported in the 5-day regimen, which calls attention to infection screening and antibiotic prophylaxis in the 10-day regimen.

Relapse after allo-HSCT for AML or MDS is the main cause of treatment failure and is associated with a very poor prognosis and a short survival (101). Reductions of immunosuppressive therapy, chemotherapy with or without infusion of DLI or second allo-HSCT are regularly proposed, but the results remain disappointing, and the incidence of GvHD after the use of DLI or Len is approximately 40% (82). Compared with traditional treatments after HSCT, novel agents need to have a good response, good tolerance and low toxicity, including HMAs. Data on preventing relapse of using single HMAs posttransplant after allo-HSCT are limited, as the antitumor effects tend to be weak. Although no direct comparison was performed, HMA-based therapies showed advantages in reducing GvHD over other traditional therapies, such as DLI, Len and some targeted drugs (**Table 3**).

**Figure 1:**
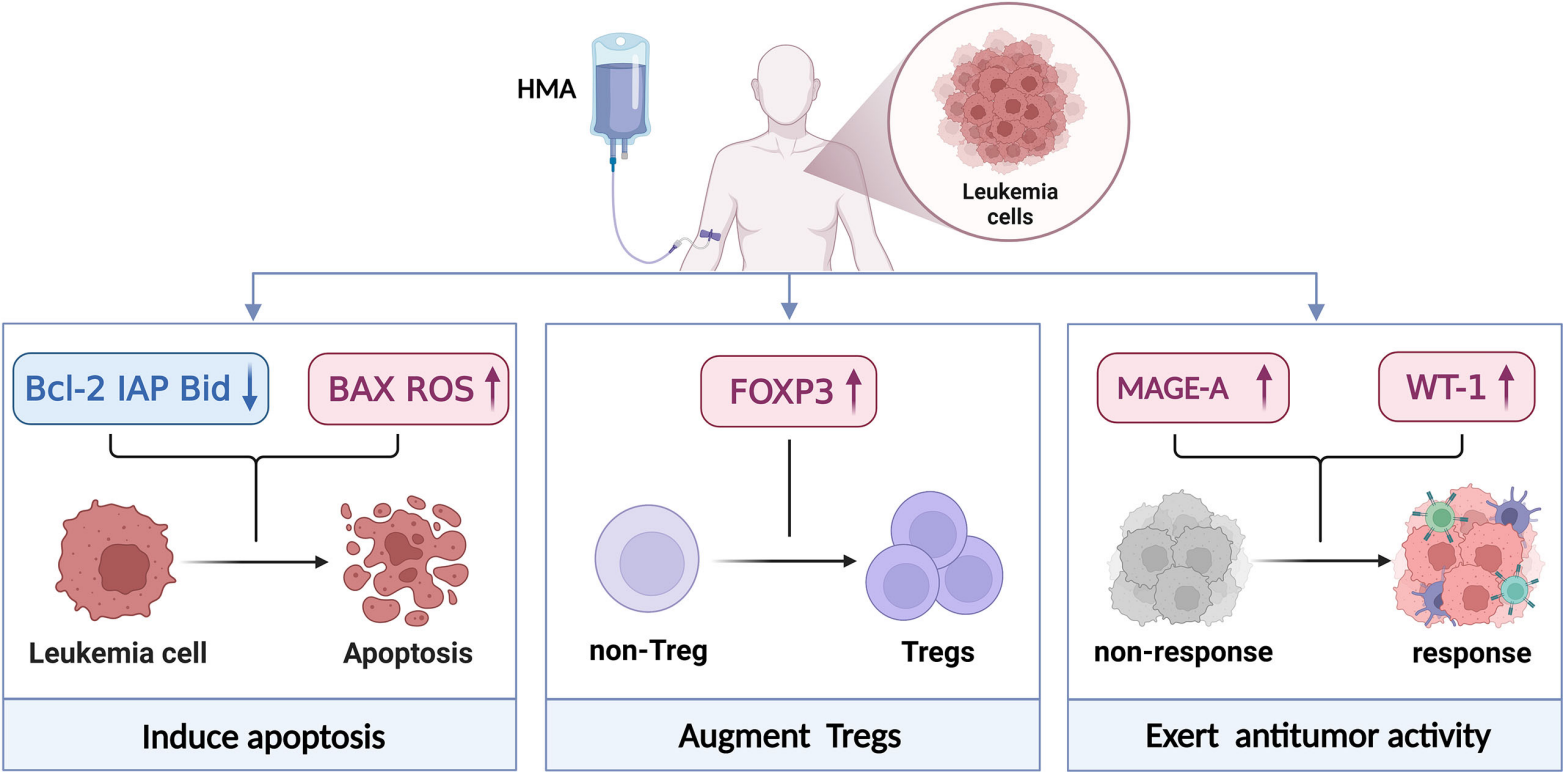
Mechanism of action of HMAs. HMAs induced apoptosis by engaging extrinsic and intrinsic apoptosis pathways and intracellular reactive oxygen species generation. This process was correlated with the downregulation of anti-apoptotic Bcl-2, IAP protein levels, the cleavage of Bid proteins, BAX activation and ROS upregulation which contributes to cell death. HMAs could mitigate GvHD by increasing the number and function (CD4^+^CD25^-^ non-Tregs convert to CD4^+^CD25^-^FOXP3^+^Tregs) of Tregs after allo-HSCT. And their suppressor function is dependent on direct contact, partially dependent on perforin 1 (Prf1). When combined with other drugs, HMAs could enhance the immunotherapy responses and sensitise immunologically recalcitrant tumours to immunotherapy. Created with BioRender.com.

The efficacy and safety of HMAs alone or HMA-based strategies to prevent and treat recurrence after allo-HSCT are different. HMAs alone have been proven to cause cell apoptosis in leukemia cells and improve Tregs conversion and expansion *in vitro*. When combined with other therapies, HMAs could potentially reverse some epigenetic silencing genes like MAGE-1 and WT-1 to exert an antitumor effect (**Figure 1**). Therefore, scientists and clinicians need to use HMAs to prevent and treat recurrence after allo-HSCT according to the following rules. 1) Patients should be administered HMAs as early as possible after allo-HSCT to maximize the efficacy, especially in patients with a relatively low leukemic burden as well as slow-growing tumors. 2) Regarding safety, HMAs have been reported to have direct cellular toxicity, especially at high doses, and it might be inappropriate to administer DLI immediately after HMAs. Oral AZA CC-486 showed beneficial effects on prolonged administration and reduced injection-site reactions (102). 3) To increase the clinical effectiveness, the combination of HMAs with synergistic therapies was more feasible than monotherapy, especially in the salvage setting.

Currently, some other epigenetic drugs have been explored in the nontransplant setting. Guadecitabine, a next-generation HMA, can reduce the degradation of DAC and prolong its exposure *in vivo*, which has been used in myeloid neoplasia and has shown promising efficacy in elderly patients with AML (103). Current studies suggested that HDACi could reduce the incidence of GvHD by inhibiting the production of proinflammatory cytokines, regulating the function of alloreactive T cells, and upregulating the function and number of regulatory T cells (76). Both preclinical and clinical studies have shown that combinations of HMAs and HDACi may exert additive or synergistic antitumor activity (57, 104–106). The limited understanding of HDACi in the transplant setting is due to a relatively small number of studies. In addition, isocitrate dehydrogenase (IDH) and bromodomain and extraterminal (BET) inhibitors have also been reported in the treatment of myeloid neoplasms (107, 108), and further studies should be carried out to elucidate the role of IDH and BET inhibitors in the HSCT setting.

**Figure 2:**
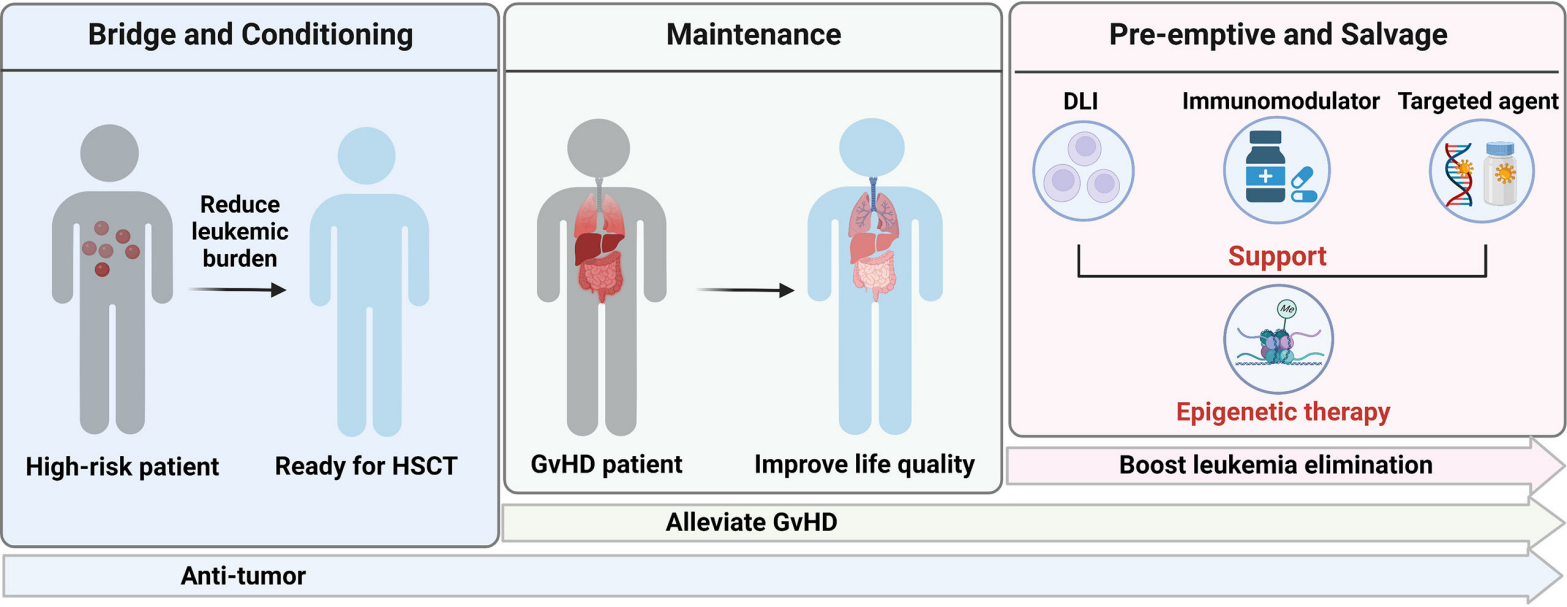
Application of epigenetic therapy in HSCT. Created with BioRender.com.

In summary, residual disease before HSCT, GvHD and relapse after HSCT remain three challenges for MDS and AML patients receiving allografts and our study highlight epigenetic therapy could be a generalist strategy in transplant-setting due to its immunologic activity and safety profile (**Figure 2**).

## Author contributions

The manuscript was conceptualized by XZ and XiaoW. GY wrote the majority of the manuscript. GY, XianW and SH contributed equally to the manuscript. The figures were designed by JW and RH and drawn by GY and SH. GY, XianW, and SH summarized the tables. All authors contributed to the article and approved the submitted version.

## Funding

This work was supported by grants from the National Natural Science Foundation of China (Nos. 81873424 and 82100235), Natural Science Foundation of Chongqing Innovation Group Science Program (No. cstc2021jcyj-cxttX0001) and Clinical Medical Research Project of Army Medical University (Major Project A, No. 2018XLC1006).

## Conflict of interest

The authors declare that the research was conducted in the absence of any commercial or financial relationships that could be construed as a potential conflict of interest.

## Publisher’s note

All claims expressed in this article are solely those of the authors and do not necessarily represent those of their affiliated organizations, or those of the publisher, the editors and the reviewers. Any product that may be evaluated in this article, or claim that may be made by its manufacturer, is not guaranteed or endorsed by the publisher.
